# A DFTB study on the electronic response of encapsulated DNA nucleobases onto chiral CNTs as a sequencer

**DOI:** 10.1038/s41598-024-61677-0

**Published:** 2024-05-11

**Authors:** Seyyed Mostafa Monavari, Nafiseh Memarian

**Affiliations:** Faculty of Physics, Semnan University, P.O. Box: 35195-363, Semnan, Iran

**Keywords:** DFTB method, Sequencing, Band gap, DNA, Biosensors, Absorption energy, Applied physics, Biological physics

## Abstract

Sequencing the DNA nucleobases is essential in the diagnosis and treatment of many diseases related to human genes. In this article, the encapsulation of DNA nucleobases with some of the important synthesized chiral (7, 6), (8, 6), and (10, 8) carbon nanotubes were investigated. The structures were modeled by applying density functional theory based on tight binding method (DFTB) by considering semi-empirical basis sets. Encapsulating DNA nucleobases on the inside of CNTs caused changes in the electronic properties of the selected chiral CNTs. The results confirmed that van der Waals (vdW) interactions, π-orbitals interactions, non-bonded electron pairs, and the presence of high electronegative atoms are the key factors for these changes. The result of electronic parameters showed that among the CNTs, CNT (8, 6) is a suitable choice in sequencing guanine (G) and cytosine (C) DNA nucleobases. However, they are not able to sequence adenine (A) and thymine (T). According to the band gap energy engineering approach and absorption energy, the presence of G and C DNA nucleobases decreased the band gap energy of CNTs. Hence selected CNTs suggested as biosensor substrates for sequencing G and C DNA nucleobases.

## Introduction

Deoxyribonucleic acid (DNA) is a molecule that contains genetic information and is composed of nucleotides. Each nucleotide consists of a nitrogenous base—adenine (A), guanine (G), cytosine (C), and thymine (T)—, a sugar molecule (deoxyribose), and a phosphate group. The arrangement of the nitrogenous bases (called as nucleobases) plays an important role in the immune system’s defense against diseases such as cancer^1,2^. In addition to the main nucleobases, there can also be some types of abnormal bases in the structure of DNA, such as methylation, alkylation, deamination, tautomerization, etc^3,4^. After the discovery of the DNA molecule, genetics researchers tried to find out how to sequence the human genome. During DNA sequencing, the primary goal is to determine the order of these nitrogenous bases along the DNA strand^5^. By knowing the gene sequence, it is possible to understand various changes in cells (genetic mutations) and different factors affecting new diseases. It is also possible to avoid the spread of diseases to future generations by preventing and finding suitable approaches^6–11^.

Biosensors are nowadays ubiquitous in different areas of healthcare. The concept of biosensors was first introduced in the early 1960s, as an “enzyme electrode” to measure the glucose concentration of diabetic patients by glucose oxidase enzyme^12^. Biosensors are devices that exhibit predictable reactions under specific conditions. The development of more accurate, smaller, and more capable biosensors has attracted the attention of researchers^13^. In general, based on the transducer, biosensors are classified into electrochemical, acoustic, thermal, mass, and optical types, each of which can measure different diagnostic parameters^14,15^. Increasing the sensitivity, efficiency, and accuracy of these biosensors requires the discovery of new materials and devices^13^.

The use of nanomaterials in biosensors, in addition to increasing its sensitivity, also reduces its response time compared to conventional diagnosis methods^16,17^. These biosensors potentially track DNA-based biomarkers for early detection of cancer, genetic disorders, and the response of the patient's body to treatment methods^18–20^. One of the candidates for biosensor fabrication is carbon nanomaterials^21–23^. Among, carbon nanomaterials, carbon nanotubes (CNTs) have unique properties and can cross biological barriers. These specific features of CNTs make them favorable to use in various fields, including: polymer nanocomposites^24,25^, encapsulating drugs/ nanoparticles/ radioactive elements^26^, as sensor substrates for sequencing DNA nucleobases^27^, biosensors for detection of SARS-CoV-2 virus spike proteins^28^, and absorption of inorganic nanoparticles Furthermore, CNTs are inflexible nonpolar candidates for encapsulation^29^ which can often be absorbed by cells without detection by the body’s immune system^30,31^. This could be very important for smart drug delivery applications^32,33^.

One method to utilize the CNTs for DNA sequencing is based on the study of electronic properties, such as band gap energy or different binding energy between nucleobases and CNTs. In this way, changing the characteristics of “CNT + nucleobases” combination leads to determination between bases and as a result the sequencing of DNA nucleobases. In this field, some theoretical studies have been done to estimate the strength of interaction between DNA bases and CNTs^34–36^. For instance, Cruz et al. have studied the encapsulation of DNA nucleobases inside CNT (16, 0) and (22, 0) by using molecular dynamics. By analyzing the thermodynamic, dynamic, and free energy, they have reported the sequence between DNA nucleobases when the bases are encapsulated in the CNTs^35^. Furthermore, experimental results indicate that pristine and Pt-labelled DNA molecules can be encapsulated inside the CNTs. It is worth noting that the CNTs should have proper chirality as well as DNA molecules should be loaded parallel to the CNT growth direction^37^.

In this research, we are looking for electronic parameters that can be used for the DNA nucleobases sequencing. We also considered DNA nucleobases encapsulation effects inside the selected chiral CNTs. Three chiral CNT (7, 6), CNT (8, 6), and CNT (10, 8), which can be synthesized experimentally, were selected. For sequencing, we examined some of the most important electronic parameters such as band structure, band gap, electron density of states (DOS), and absorption energy. Also, the possibility of using selected CNTs as biosensor substrates is investigated.

## Calculation method

**Figure 1:**
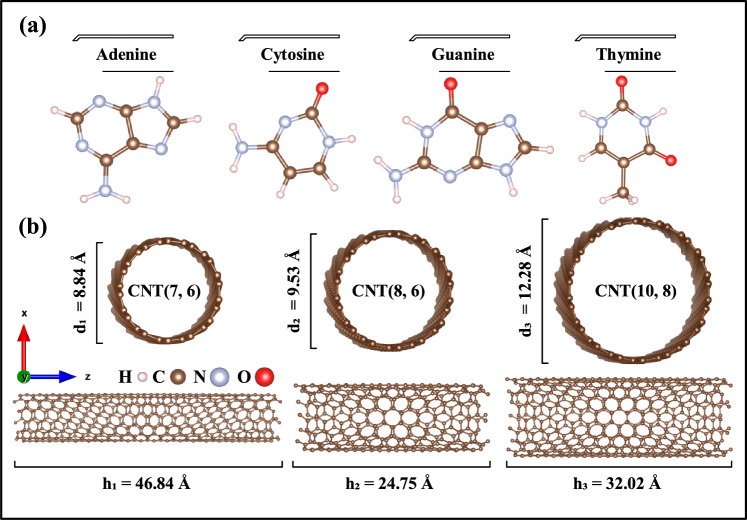
Optimized structures of (**a**) DNA nucleobases and (**b**) pristine selected chiral CNTs.

**Figure 2:**
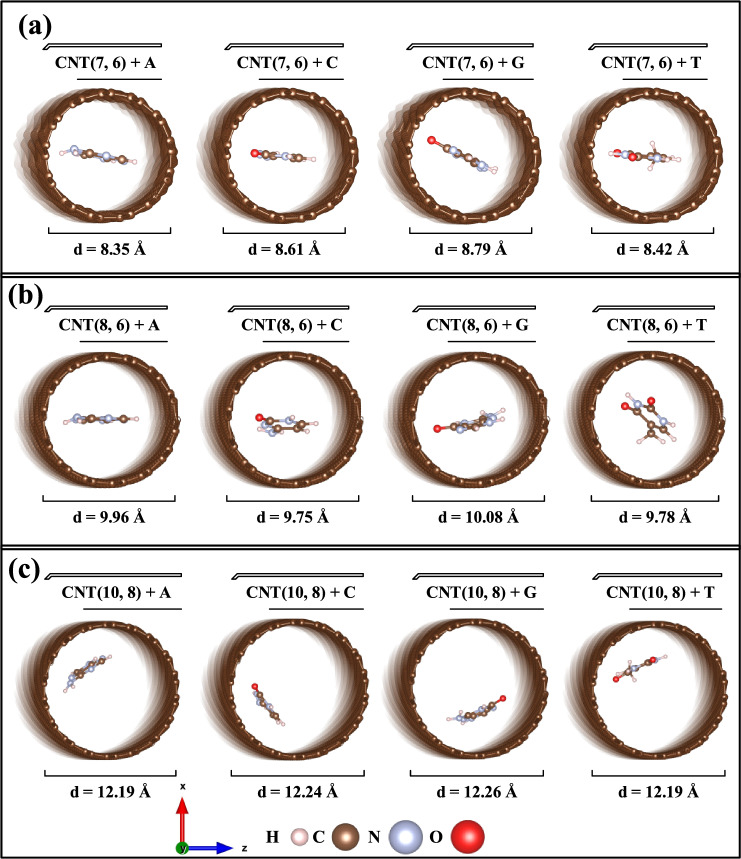
(**a**–**c**) Optimized structures for encapsulated DNA nucleobases on the inside of CNT (7, 6), CNT (8, 6), and CNT (10, 8), respectively.

The electronic and absorption properties have been studied by using DFTB method. Calculations were done with the DFTB+ package^38–40^. Calculations related to the solution of many-body equations have been performed using the semi-empirical Slater–Koster CHNO basis set with the generalized gradient algorithm (GGA)^41^. After performing the convergence calculations related to energy and forces, by values of 0.05 kcal/mol/Å and 0.01 kcal/mol, respectively. Also, K-point mesh and cutoff energy were chosen as 1 × 1 × 50 and 300 Ry, respectively. In structural optimization, the physiological body temperature of 300 K, the boundary condition (PBC) along the CNTs growth orientation (Z), and the effect of van der Waals (vdW) interactions have been considered^42^. In the optimization process, we designed each of the DNA nucleobases parallel to the CNT growth orientation and in the center of the simulation supercell. By applying self-consistent calculations, energy, forces, and K-point convergences were performed before structural optimization calculations, and the necessary converged values were selected for each structure. In the next step, the dimensions of the simulation supercell and the position of the atoms have been optimized. Finally, in order to calculate electronic and transport properties, the results obtained in the structural optimization stage have been used. The information obtained from these methods predicts the equilibrium properties of nanostructures. Calculations of electronic properties were performed at room temperature (300 K). Optimized structures of DNA nucleobases, chiral CNTs (CNT (7, 6), CNT (8, 6), and CNT (10, 8)), and encapsulated CNTs with DNA nucleobases are shown in Figs. 1 and 2.

## Results and discussion

The use of DFTB method is very convenient for systems containing the high number of atoms and the possibility of using DFT is associated with heavy computational costs. First, we designed the DNA nucleobases and pristine chiral CNT (7, 6), CNT (8, 6), and CNT (10, 8) individually in the simulation unit cell. Then we started energies and forces convergence for structural optimization steps. Then, we encapsulate the DNA nucleobases parallel to the CNTs growth axis (Z) in the center of CNTs. In this way, the encapsulation process of DNA nucleobases was studied by selected pristine chiral CNTs. For this purpose, the position of the atoms inside the unit cell and the dimensions of the unit cell were optimized by considering the convergence conditions mentioned in the computational details section. By studying the total energies of pristine cases (CNTs and DNA nucleobases) and encapsulated systems, we investigate the phenomenon of physical absorption. The values of absorption energy and band gap energy differences for the encapsulated system have been obtained by using Eq. 1.

1 $$ \begin{gathered} \Delta E_{B} = \, E\,_{(DNA\,nucleobases)} + \, E\,_{(CNT)} - E\,_{(Encapsulate \, system)} \hfill \\ \Delta E_{gap} = \, E_{gap} \,_{(CNT)} - \, E_{gap} \,_{(Encapsulate \, DNA\,nucleobases)} \hfill \\ \end{gathered} $$

where E (DNA nucleobases) is the total energy of pristine DNA nucleobases, E (CNT) is the total energy of pristine CNTs, and E (Encapsulate system) is related to the total energy of encapsulated systems. The results of absorption energy are reported in Table 1. In addition to the adsorption energy, we investigate properties that can be used to indicate the sequence between DNA nucleobases in encapsulated systems. These properties include band gap energy (difference value between the highest occupied energy level in the valence band and the lowest unoccupied energy level of the conduction band), band structure, DOS, and highest occupied molecule orbital (HOMO) and lowest unoccupied molecular orbital (LUMO) surfaces. For sequence between encapsulated DNA nucleobases on the inside of CNTs, we emphasized on band gap energy engineering. Sequencing parameters containing the number of atoms in the simulation unit cell (N), band gap energy (E_gap_), band gap energy differences (ΔE_gap_), and absorption energy (ΔE_B_) are listed in Table 1.

**Table 1:** Sequencing parameters for pristine CNTs and encapsulated DNA nucleobases systems.

Structures	N	E_gap_ (eV)	ΔE_gap_ (eV)	ΔE_B_ (eV)
CNT (7, 6)	508	0.789 – (0.821^28^, 1.105(Exp.)^43^)	–	–
CNT (8, 6)	296	0.747	–	–
CNT (10, 8)	488	0.580	–	–
CNT (7, 6) + A	523	0.787	0	0.98
CNT (7, 6) + C	521	0.605	0.18	1.04
CNT (7, 6) + G	524	0.578	0.21	1.17
CNT (7, 6) + T	523	0.788	0	1.40
CNT (8, 6) + A	311	0.747	0	1.55
CNT (8, 6) + C	309	0.559	0.19	1.25
CNT (8, 6) + G	312	0.505	0.24	1.69
CNT (8, 6) + T	311	0.747	0	1.68
CNT (10, 8) + A	503	0.580	0	1.31
CNT (10, 8) + C	501	0.394	0.19	0.81
CNT (10, 8) + G	504	0.349	0.23	1.43
CNT (10, 8) + T	503	0.580	0	1.19

The results indicate that there are similarities in the behavior of DNA nucleobases in selected chiral CNTs. In such a way that the most changes in the band gap energy happened for the encapsulation of G and C nucleobases, while despite the interactions of A and T nucleobases with selected CNTs, the changes in the band gap energy are very weak and we predict that these DNA nucleobases to make changes in the optical properties of CNTs. CNTs based on their chiral indices can be divided into three electronic categories (metals, insulators, and semiconductors)^44–46^. Due to band gap energy values for chiral CNT (7, 6), CNT (8, 6), and CNT (10, 8), they are classified as semiconductors. In addition, by increasing the diameter of the CNTs from 8.84 to 12.28 Å, the band gap energy has decreased due to the quantum confinement effect^47,48^.

Furthermore, Similarities have been observed in some of the electronic properties of CNT (7, 6) when DNA nucleobases were applied around its external environment and encapsulated cases. Such that the band gap energy differences (ΔE_gap_) for both cases (encapsulated and outer side environment) follows the pattern: CNT (7, 6) + G > CNT (7, 6) + C > CNT (7, 6) + A > CNT (7, 6) + T. The results revealed that in the encapsulated cases the ΔE_gap_ decreased from 0.48 to 0.21 eV for G nucleobase and from 0.384 to 0.18 eV for C nucleobase. In addition, the presence of A and T nucleobases in the outside environment region and encapsulated inside of CNT (7, 6) did not cause significant change in the band gap energy values^27^.

As can be noted in Table 1, since the binding energy values for all the encapsulated structures are larger than 0.65 eV, it can be said that the strength of the hydrogen bond is high. So, strong physical adsorption has occurred between DNA nucleobases and CNTs^49–52^. The interaction between the π-orbitals of DNA nucleobases and chiral CNTs plays a crucial role during the physisorption process of DNA nucleobases on chiral CNTs. Among the studied cases, encapsulated G nucleotide base in CNT (8, 6) and encapsulated C nucleotide base in CNT (10, 8) showed the highest and lowest physisorption with absorption energy values of 1.69 eV and 0.81 eV, respectively.

**Figure 3:**
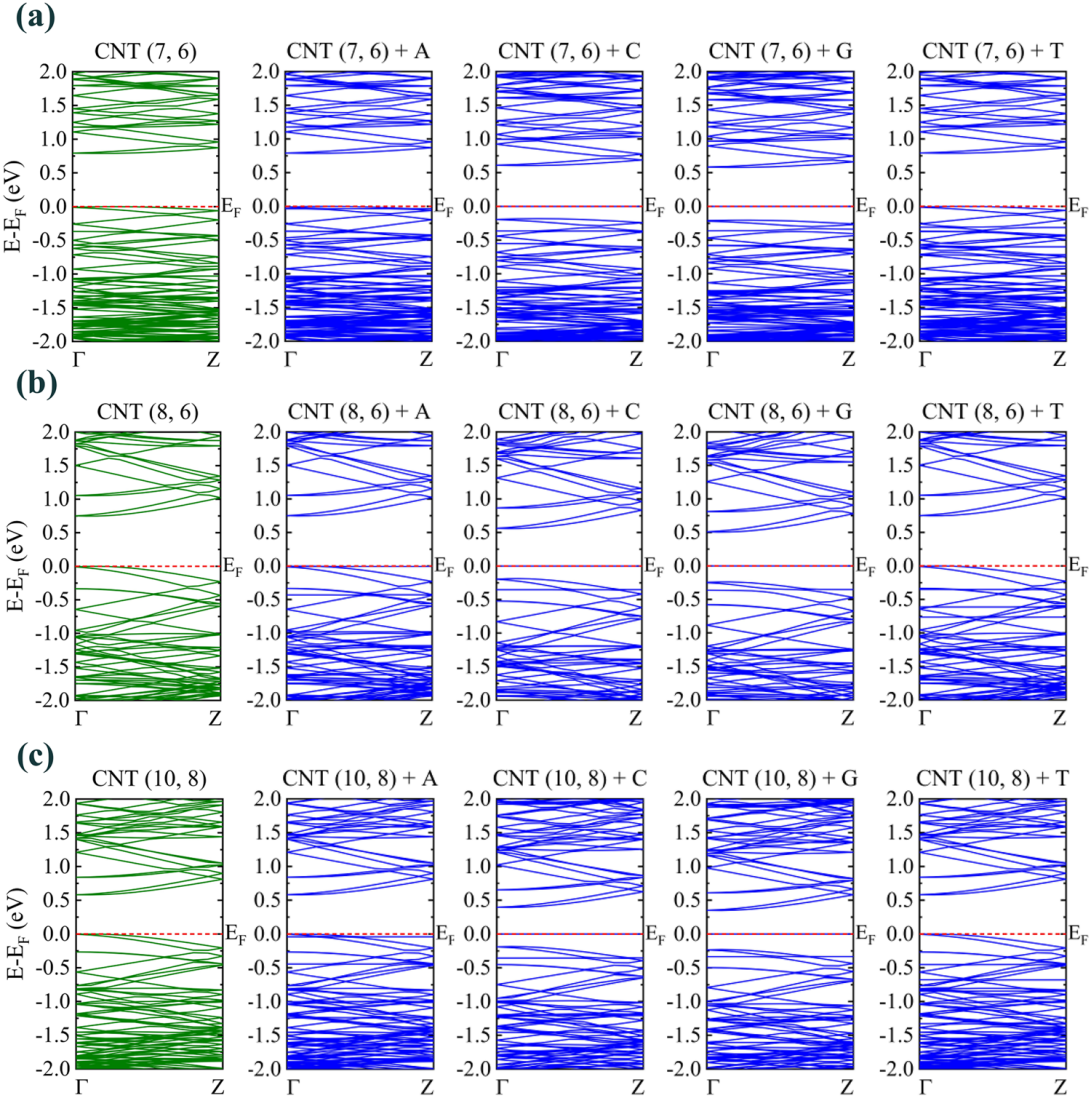
Effects of DNA nucleobases encapsulation in the band structure of (**a**) CNT (7, 6), (**b**) CNT (8, 6), and (**c**) CNT (10, 8). green bands correspond to pristine CNTs and blue bands correspond to encapsulated systems.

Next, we investigated the band structure, DOS, and HOMO–LUMO surfaces for pristine and encapsulated systems. The band structure of pristine CNTs and the encapsulated DNA nucleotide base systems are shown in Fig. 3 in green and blue colors, respectively. According to the CNTs growth direction (Z), the band structure in the first Brillouin zone is drawn in the path of Γ to Ζ, and Fermi energy is set to zero. The band gap energies of all the studied systems are direct. The presence of DNA nucleobases has caused an increase in the number of bands in the chosen energy ranges (− 2 to 2 eV). So we expect the DOS to increase. Furthermore, when G and C DNA nucleobases are encapsulated on the inside of CNTs, the HOMO level in the valence band and the LUMO level in the conduction band become closer to each other and the band gap energy of the encapsulated systems decreased, as ΔE_gap_ listed in Table 1.

**Figure 4:**
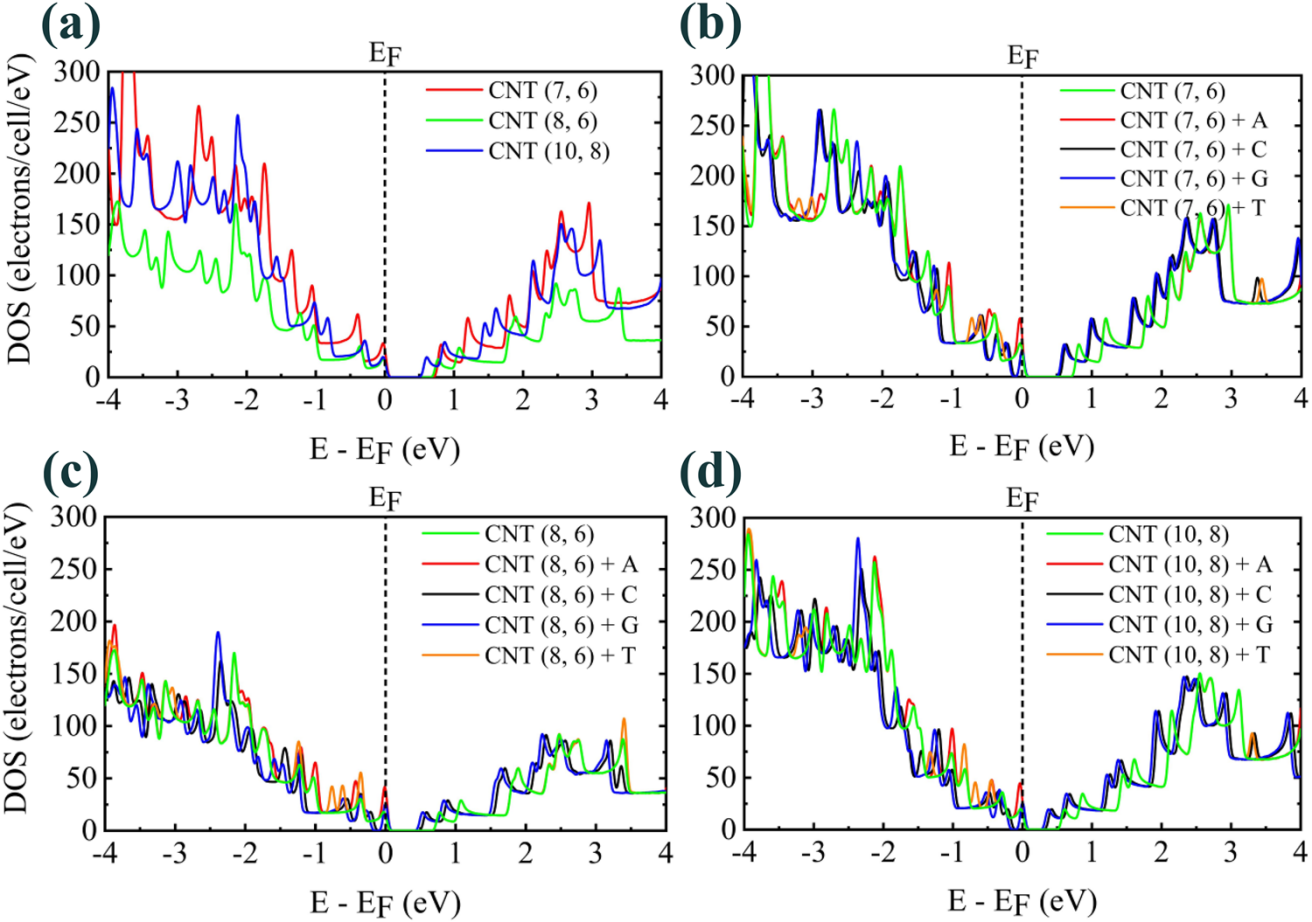
DOS for (**a**) pristine CNTs (**b**–**d**) encapsulated DNA nucleobases on the inside of chiral CNT (7, 6), CNT (8, 6), and CNT (10, 8), respectively. Fermi energy is set to zero.

Since the number of electron states in valence and conduction bands is very large, the concept of DOS is used to express the number of these states in each specific energy level. The DOS was obtained by the smearing integration method and choosing the smearing value of 0.03 eV (smearing = 0.03 eV) with the 300 number of points per eV. The increase in diameter of selected CNTs and the presence of DNA nucleobases inside selected chiral CNTs have caused changes in DOS (Fig. 4). CNT (7, 6), despite having the smallest diameter compared to the others, has the highest DOS due to having a large number of atoms in the unit cell (Fig. 4a).

The number of atoms for DNA nucleobases is in the pattern of N(G) > N(A) = N(T) > N(C). So, it can be expected that the DOS for encapsulated systems follows the aforementioned pattern. (Fig. 4b–d). In fact, the encapsulation of DNA nucleobases has led to the sequencing between these four bases by all of the selected chiral CNTs.

**Figure 5:**
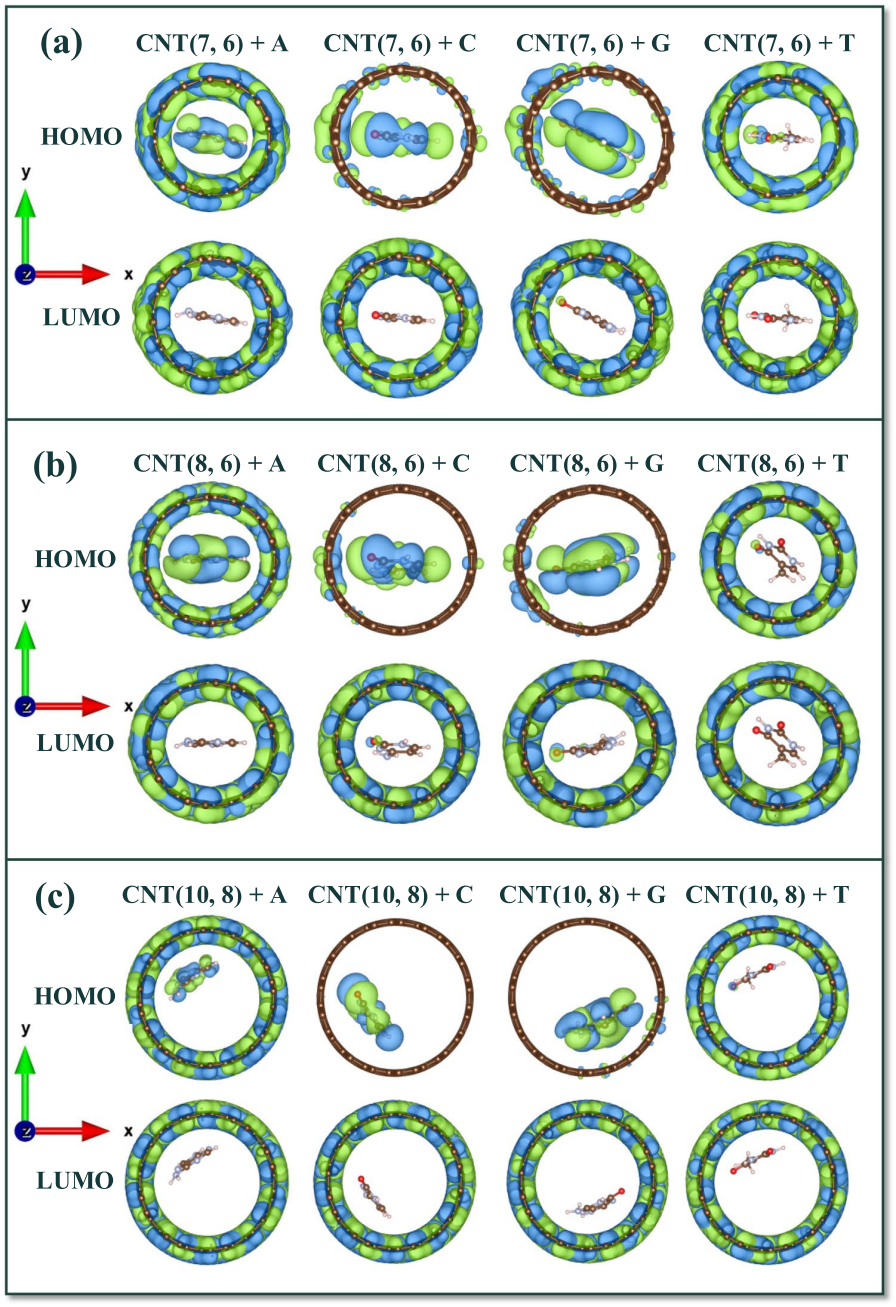
HOMO and LUMO isosurface for encapsulated DNA nucleobases on the inside of (**a**) CNT (7, 6), (**b**) CNT (8, 6), and (**c**) CNT (10, 8). Isosurfaces are plotted with 0.008 e/Å^3^ isovalues. Positive and negative electric charges are shown in green and blue colors, respectively.

Finally, HOMO and LUMO surfaces have been considered for the sequencing purpose and presented in Fig. 5. According to the dimensions of the DNA nucleobases and the space localization on the inside of CNTs for substitution of nucleobases, van der Waals (vdW) interactions have caused different behaviors in the HOMO and LUMO surfaces. The difference in HOMO and LUMO surfaces is due to the presence of electronegative atoms in the structure, such as oxygen and nitrogen, and π-orbitals interactions. As shown in Fig. 5, the influence of DNA nucleobases on the HOMO surfaces of CNTs is G > C > A > T. On the other hand, it can be said that non-bonded electron pairs interacted with CNT carbon atoms, causing an inter-gap level in the band gap region. Therefore, the band gap energy of CNTs + DNA nucleobases has decreased. In this analysis also, the presence of electronegative atoms in G and C nucleobases has caused more significant changes in HOMO and LUMO surfaces. The results showed that in the LUMO surfaces of all the encapsulated systems, electronic charges are suppressed on the selected CNTs. The positive and negative electronic charge densities on the DNA nucleobases are very weak and more concentrated on the CNT carbon atoms. While, for HOMO surfaces, a different trend can be observed. At the HOMO surfaces, the charge transfer from the CNTs to the DNA nucleobases and dipole interactions between them are observed. These interactions are stronger for G and C DNA nucleobases.

## Conclusion

This research aimed to predict the behavior of DNA nucleobases when they were encapsulate by chiral CNTs. For this purpose, three CNT (7, 6), CNT (8, 6), and CNT (10, 8) which also could be synthesized experimentally have been selected. Here, the sequence between DNA nucleobases when encapsulated on the inside of CNTs using electronical properties is studied. The study was conducted theoretically using the DFTB method due to the large number of atoms in the unit cell and the need to use semi-empirical approximations. After convergences and structural optimization, electronic properties such as binding energy, hydrogen bond strength, band structure, band gap energy, DOS, and HOMO–LUMO orbital surfaces have been investigated. Our results showed that CNT (8, 6) has better separation and sequencing between DNA nucleobases than CNT (7, 6) and CNT (10, 8). The results revealed that CNTs performed better in the sequence of G and C DNA nucleobases. This could be due to the presence of non-bonding electrons in high electronegative elements in G and C nucleobases, so they are able to reduce the band gap energy of CNTs. Reducing the band gap energy is happened through the creation of inter-gap levels. These band gap changes can lead to the generation of electrical pulses, and in this way, our selected CNTs suggested as biosensor substrates for G and C nucleobases sequencing.

## Data Availability

The datasets used and/or analyzed during the current study are available from the corresponding author upon reasonable request.
